# Serum Free Light Chains in Common Variable Immunodeficiency Disorders: Role in Differential Diagnosis and Association With Clinical Phenotype

**DOI:** 10.3389/fimmu.2020.00319

**Published:** 2020-03-31

**Authors:** Riccardo Scarpa, Federica Pulvirenti, Antonio Pecoraro, Alessandra Vultaggio, Carolina Marasco, Roberto Ria, Sara Altinier, Nicolò Compagno, Davide Firinu, Mario Plebani, Marco De Carli, Andrea Matucci, Fabrizio Vianello, Angelo Vacca, Giuseppe Spadaro, Isabella Quinti, Carlo Agostini, Cinzia Milito, Francesco Cinetto

**Affiliations:** ^1^Department of Medicine—DIMED, University of Padova, Padova, Italy; ^2^Rare Disease Referral Center, Internal Medicine 1, Ca' Foncello Hospital, ULSS2 Marca Trevigiana, Treviso, Italy; ^3^Department of Infectious Diseases and Internal Medicine, University Hospital Policlinico Umberto I, Rome, Italy; ^4^Department of Translational Medical Sciences, Center for Basic and Clinical Immunology Research, University of Naples Federico II, Naples, Italy; ^5^Immunoallergology Unit, Department Medical-Geriatric, AOU Careggi, Florence, Italy; ^6^Department of Biomedical Sciences and Human Oncology, School of Medicine, University of Bari Aldo Moro, Bari, Italy; ^7^Department of Laboratory Medicine of the University Hospital of Padova, Padova, Italy; ^8^Hematology and Clinical Immunology, Department of Medicine—DIMED, University of Padova, Padova, Italy; ^9^Department of Medical Sciences and Public Health, University of Cagliari, Cagliari, Italy; ^10^Department of Internal Medicine, Azienda Sanitaria Universitaria Integrata di Udine, Udine, Italy; ^11^Department of Molecular Medicine, Sapienza University of Rome, Rome, Italy

**Keywords:** serum free light chains, common variable immunodeficiency disorders (CVID), lymphoproliferative disease (LPD), diagnostic marker, immunoglobulin, clinical phenotype, non-infectious complications

## Abstract

We report on an observational, multicenter study of 345 adult CVID patients, designed to assess the diagnostic value and the clinical association of serum free light chain (sFLC) pattern in Common Variable Immunodeficiency disorders (CVID). Sixty CVID patients were tested twice in order to assess intraindividual variability of sFLC. As control groups we included 138 patients affected by undefined primary antibody defects (UAD), lymphoproliferative diseases (LPDs), and secondary antibody deficiencies not related to hematological malignancies (SID). CVID patients presented lower κ and λ chain concentration compared to controls, showing low intraindividual sFLC variability. On the basis of the sFLC pattern, patients were classified into four groups: κ−λ+, κ+λ−, κ−λ−, κ+λ+. The most common pattern in CVID patients was κ−λ− (51%), followed by κ−λ+, (25%), κ+λ+ (22%), and κ+λ− (3%). In UAD, LPD, and SID groups κ+λ+ was the most common pattern observed. By analyzing the possible association between sFLC patterns and disease-related complications of CVID, we observed that patients belonging to the κ−λ− group presented more commonly unexplained enteropathy compared to the κ+λ+ group and showed higher frequency of bronchiectasis and splenomegaly compared to both the κ−λ+ and κ+λ+ patients. When compared to the other groups, κ−λ− had also lower serum IgG, IgA, and IgM concentrations at diagnosis, lower frequency of CD27+IgD–IgM– switched memory B cells, and higher frequency of CD21^low^ B cells, receiving earlier CVID diagnosis. Thus, lower levels of sFLC might be an epiphenomenon of impairment in B cell differentiation, possibly leading κ−λ− patients to a higher risk for bacterial infections and chronic lung damage. Based on these results, we suggest adding sFLC assay to the diagnostic work-up of hypogammaglobulinemia and during follow-up. The assay may be useful to differentiate CVID from other causes of hypogammaglobulinemia and to early detect monoclonal lymphoproliferation occurring over years. Moreover, since the sFLC pattern seems to be related to disease phenotypes and clinical manifestations of CVID and after confirmation by further studies, sFLC assay might be considered a promising prognostic tool for identifying patients at higher risk of developing enteropathy and chronic lung damage or splenomegaly. This will allow designing a tailored follow-up for CVID patients.

## Background

Common variable immunodeficiency disorder (CVID) is the most common symptomatic primary antibody deficiency (PAD) diagnosed in adulthood, affecting 1:25,000–1:50,000 patients in western countries (1, 2). Due to the lack of confirmative test, diagnostic criteria include low circulating immunoglobulin count (decrease in IgG and IgA, +/– IgM), exclusion of other causes of hypogammaglobulinemia, and a list of other immunologic parameters (e.g., poor response to vaccination, low percentage of switched memory B cells) that can differentiate CVID from unclassified antibody deficiency (UAD) (https://esid.org/Education/Diagnostic-Criteria-PID). From a clinical point of view, manifestations of CVID are extremely heterogenous, leading to different clinical phenotypes and prognosis, with patients without complications showing better survival (3, 4).

Immunoglobulins are formed by a double pair of identical heavy and light chains, these latter being kappa (κ) or lambda (λ) chains. Free light chains are usually detectable in the serum (sFLC) due to an excess in production (around 40%) compared to heavy chains (5). A serum FLC (sFLC) nephelometric assay has been developed almost 20 years ago to accurately measure the amounts of circulating serum free κ and λ light chains (6). Serum and/or urine testing for monoclonal free light chains, together with the presence of an M-protein (paraprotein), is critical to the diagnosis of multiple myeloma (MM). In rare cases, malignant plasma cells, such as in MM and related disorders, secrete only a clonal light chain without any identifiable heavy chain. Monoclonal increase of κ or λ chain serum levels, leading to an abnormal serum FLC ratio (FLCr), may thus reveal the presence of a non-secretory myeloma in case a paraprotein is absent, thus reducing diagnostic delay (7). In the case of immunoglobulin light chain (AL) amyloid deposition, sFLC assay has been shown to be more sensitive for diagnostic purposes than conventional serum electrophoresis or immunofixation (5). Besides plasma cells dyscrasias, a significant imbalance in sFLC secretion has been suggested as a sensitive indicator of B-cell clonality and as a prognostic marker in chronic lymphocytic leukemia (B-CLL), monoclonal B-cell lymphocytosis (MBL), and some B-cell non-Hodgkin lymphomas (NHL) (8–10). Apart from revealing the presence of a clonal, κ- or λ-restricted cell population, sFLC absolute values correlate with disease activity and clinical outcome in patients with plasma cell malignancies (11). Moreover, increased sFLC levels with a normal FLCr have been suggested as a useful marker of the polyclonal B-cell activation and/or expansion sustaining the activity of different chronic inflammatory and autoimmune conditions (12). Finally, an impaired sFLC catabolism may be an indirect signature of a progressive renal injury (13).

Being a sensitive, simple, and reproducible indicator of B-cell biologic activity, the potentiality of sFLC assay has been more recently explored in the context of primary B-cell impairments/deficiency, including PAD and particularly CVID.

In 2012, Unsworth et al. first reported in a small cohort of PAD patients extremely low levels of κ and/or λ chains (below the limits of reliable detection) in 19/20 cases, mostly CVID, leading to impaired or non-calculable serum FLCr. Thus, they suggested that a suspicious κ/λ ratio, usually due to very low absolute quantities of at least one sFLC, most likely underlies the disease-related B-cell dysfunction, rather than a B lymphocyte clonality in this specific setting (14).

We subsequently reported a possible role of sFLC in the differential diagnosis between a primary and secondary hypogammaglobulinemia, suggesting their possible role in risk stratification of adults with CVID, in terms of peculiar biological characteristics, clinical behavior, and prognosis (15).

Recently, sFLC have been described as a marker for differentiation of PAD, with a high specificity but limited sensitivity for CVID. A possible prognostic and therapeutic relevance has also been hypothesized, reporting significant results in a single center cohort of 81 CVID patients (16).

Finally, rare cases of primary immunoglobulin κ light chain defects have been reported since 1972, whose genetic basis has been extensively explored only in two cases (17). κ chain deficiency is included in the 2017 IUIS Phenotypic Classification for Primary Immunodeficiencies between PADs, reported as asymptomatic (18).

Herein we report the results of an observational multicenter study of sFLC levels in a cohort of 345 CVID patients. sFLC levels have been correlated with clinical and laboratory features of CVID. sFLC pattern of CVID patients has been compared to that of three different cohorts of non-CVID patients referred due to a first recognition of hypogammaglobulinemia, further characterized as secondary to lymphoproliferative diseases (LPDs), secondary to protein loss or medications (SID) or PAD other than CVID (unclassified antibody defects, UAD). Our findings suggest a diagnostic and potential prognostic role of sFLC assay that might be taken into account when designing personalized follow-up strategies and support its inclusion in the diagnostic work-up of CVID and other forms of hypogammaglobulinemia.

## Methods

### Study Population

We enrolled 345 adult patients (>18 y.o.) with a diagnosis of CVID (http://esid.org/Working-Parties/Registry/Diagnosis-criteria). All patients were regularly followed in inpatient and daycare settings by University Hospitals working as referral centers for adult primary immune deficiencies in Rome, Naples, Padua, Udine, Florence, and Bari and included in the IPINET Italian Registry for CVID. Exclusion criteria included inability or unwillingness to provide written informed consent. As control groups, we also included in the analysis 59 unclassified antibody defects (UAD) and 79 patients initially referred to the Padua Centre due to hypogammaglobulinemia, subsequently diagnosed as lymphoproliferative diseases (LPDs, *n* = 41) or as secondary antibody deficiencies unrelated to hematological malignancies (SID, *n* = 38). For UAD definition we used the criteria provided by the ESID registry, including patients with clinical features of PAD, marked decrease of at least one Ig isotype/IgG subclass or failure of IgG response to vaccines, who did not fit any other working definition (http://esid.org/Working-Parties/Registry/Diagnosis-criteria). All patients enrolled provided their informed consent. All the patients of the control groups underwent sFLC analysis for diagnostic purposes at the Department of Laboratory Medicine of the University Hospital of Padova. The Ethical Board of Padua approved this study, that was performed in accordance with the Good Clinical Practice guidelines, the International Conference on Harmonization guidelines, and the most recent version of the Declaration of Helsinki (15).

### Study Design

Observational, multicenter study to assess the diagnostic and prognostic value of FLC in CVID. Once the informed consent form was signed, the investigator reported participant's demographic, clinical, and laboratory data in the case report form. A set of variables was recorded for each patient including gender, date of birth, date of detection of hypogammaglobulinemia, immunoglobulin serum levels at diagnosis. Only for CVID patients we collected clinical phenotype according to the Chapel et al. proposal (19). CVID-associated conditions (cytopenia, unexplained persistent proliferation, unexplained persistent enteropathy, bronchiectasis, splenomegaly, cancer, autoimmunity, GLILD) and B-cell phenotype performed according to Wehr et al. were also registered (20). At the time of the enrollment, the serum sample for FLC assay collected during routine blood tests was then sent to the Department of Laboratory Medicine of the University Hospital of Padova as referral center (21). A second determination of sFLC concentration was available for 60 patients, with samples collected 24 ± weeks after the first time, and results were analyzed in order to assess the intraindividual variability of sFLC assay. Only patients with at least one available measurement of sFLC were finally included in the database for data analysis. CVID patients were all on immunoglobulin replacement treatment (IgRT). We included patients independently on IgRT since it has been previously demonstrated that sFLC concentration is not affected by Ig replacement (15, 16).

#### sFLC Assessment

According to standard diagnostic procedure, serum samples were stored at −80°C until FLC assay was performed. FLC (Freelite^TM^ κ and λ, Binding Site, UK) assay was applied on a BNII Nephelometer (Siemens) (22). Previously established reference ranges were used [4.52–22.33 and 4.84–21.88 mg/L for κ FLC and λ FLC, respectively and 0.44–2.67 for FLC ratio (FLCr)] (6, 21). On the basis of sFLC concentration, patients with CVID were classified into four groups: in three groups κ (κ−λ+ pattern), λ (κ+λ− pattern), or both (κ−λ− pattern) light chains were reduced or undetectable; in the fourth group they were normal (κ+λ+). Patients with more than one (previous or subsequent) available sFLC measurement did not show any significant change in pattern over time, as previously reported, unless in concomitance with a new onset of hematological malignancy (15).

#### Statistical Analysis

Patient demographics and clinical characteristics were summarized by frequencies and percentages, with means and standard deviations or median and 5–95th centile where appropriate. Comparisons of continuous parameters between treatment groups were calculated with a *t*-test if normally distributed and with a Mann–Whitney *U*-test if not normally distributed. Normal distribution of the data was evaluated by Kolmogorov–Smirnov test. Differences in frequencies between groups were calculated using the Fisher's exact test. Comparison between continuous parameter and sFLC concentration was assessed by simple linear regression analysis. Intraclass correlation coefficient (ICC) was used to evaluate intraindividual variability of both κ and λ chain serum concentration between the two available assessments. ICC values between 0.4 and 0.6, 0.6, and 0.8 or ≥0.8 were considered to indicate moderate, good, or very good agreement, respectively (23). Data were analyzed by using group sequential testing that allowed “spending” a little of the α value at each interim analysis such that the total type I error did not exceed 0.05 at the end of the study. Statistical analyses were performed with the statistical package SPSS (IBM SPSS Statistics for Windows, version 25.0; IBM, Armonk, NY).

## Results

### Baseline Characteristics

sFLC concentrations were measured in 345 CVID patients and in 138 controls (59 UAD, 41 LPDs, and 38 SID subjects). The characteristics of the cohorts are recapitulated in Table 1. The mean age of CVID at study enrollment was 49.4 ± 14.3 years, lower than what was recorded in UAD (53.6 ± 16.8 years, *t*-test, *p* = 0.050), LPDs (67.2 ± 11.2 years, *t*-test, *p* < 0.0001), and SID (56.1 ± 14.9, *t*-test, *p* = 0.016). As expected, CVID patients also displayed lower immunoglobulin serum levels at diagnosis of hypogammaglobulinemia in comparison to the other groups (Table 1). Diagnosis for patients classified as LPDs and SID are detailed in Table S1.

**Table 1:** Demographics and immunoglobulin serum levels at diagnosis in the cohort included in the analysis.

	**CVID (*n* = 345)**		**UAD (*n* = 59)**		**LPDs (*n* = 41)**		**SID (*n* = 38)**	
Age, years, mean (SD)	49.4	(14.3)	53.6	(16.8)^*^	67.2	(11.2)^****^	56.1	(14.9)^***^
Sex (female), *n* (%)	169	(49)	36	(62)	48	(40)	17	(55)
Immunoglobulin serum level at diagnosis of hypogammaglobulinemia								
IgG, g/L, median (IQR)	3.1	(1,9–3.4)	5.9	(5.2–6.7)^****^	5,3	(4.2–8.2)^****^	5.9	(5.2–6.7)^****^
IgA, g/L, median (IQR)	0.1	(0.1–0.3)	1.2	(0.9–1.6)^****^	0.4	(0.2–1.3)^****^	1.3	(1.0–1.8)^****^
IgM, g/L, median (IQR)	0.2	(0.1–0.4)	0.9	(0.4–1.1)^****^	0.3	(0.4–1.1)	0.5	(0.3–1.0)^****^

Levels of significance for comparison with CVID are:

* *P ≤ 0.05*,

*** *P ≤ 0.001*,

**** *P ≤ 0.0001*.

*Continuous variables were analyzed by t-test if normally distributed (age) or by u-test (Ig serum levels); sex was analyzed by Fisher's test*.

As shown in Table 2, 52% of CVID patients displayed an infection-only phenotype, as defined by Chapel et al. (19), whereas at least one CVID-related complication was found in the remaining subjects, namely cytopenia (20%), unexplained lymphoproliferation (33%), unexplained enteropathy (14%). Thirty-three percent of participants have had at least one autoimmune complication and 15% had a past medical history of cancer.

**Table 2:** Clinical characteristics of 345 CVID patients enrolled.

**Chapel phenotype, n (%)**		
Infection only	177	(52)
Cytopenia	68	(20)
Lymphoproliferation	113	(33)
Enteropathy	48	(14)
**CVID-related complications, n (%)**		
Splenomegaly	151	(44)
Bronchiectasis	105	(31)
Autoimmunity	114	(33)
ITP/AHE	38	(13)
Psoriasis	14	(4)
Vitiligo	15	(4)
Celiac disease	10	(3)
Other	44	(13)
GLILD	28	(8)
Malignancies	53	(15)
Lymphomas	16	(5)
Gastric cancer	13	(4)
Breast cancer	4	(1)
Colorectal cancer	4	(1)
Other	17	(5)

### Serum Free Light Chains and IgA and IgM Serum Levels

**Figure 1:**
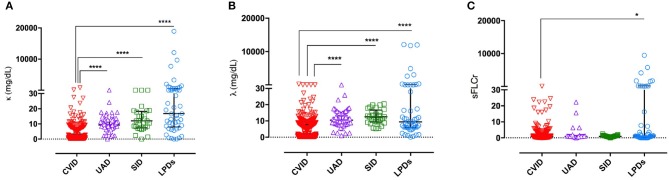
sFLC levels in the differential diagnosis of hypogammaglobulinemia. CVID patients displayed lower of κ chains serum levels **(A)** and lower λ chains serum levels **(B)** in comparison to UAD, SID and LPDs groups. CVID had also lower FLCr in comparison to LPDs **(C)**. Individual values, median, and IQR are represented. Levels of significance for comparison with CVID group by u test: *P ≤ *0.05*, ****P ≤ 0.0001.

In CVID, the median sFLC value was 2.0 mg/L (IQR 0.3–9.1) for κ chains, and 6.2 mg/dl (IQR 0.0–10.2) for λ chains, and the FLCr was 0.9 (IQR 0.6–1.4). As shown in Figure 1, CVID patients displayed lower κ and λ chain serum levels in comparison to UAD, SID, and LPDs (*u*-test *p* < 0.0001 for all comparisons). Sixty CVID patients tested twice for sFLC concentration presented a very good intraindividual agreement between the first and the second determination of both κ and λ chains (ICC 0.9 and 0.8, respectively).

CVID group showed lower serum FLCr in comparison to LPDs (*u*-test, *p* = 0.039, Figure 1C). However, 142 (41%) of CVID participants displayed an unbalanced FLCr. Among those, one patient showed very high concentration of κ chains (811 mg/L) with a FLCr of 62.9, and he was diagnosed with NHL few weeks after the study enrollment; a second patient presented a similar history. In all other patients, the very low/undetectable concentration of at least one sFLC was the reason for κ and λ chain imbalance, in agreement with previous findings (14). On the contrary, 82% of patients in the LPDs group had unbalanced FLCr due to an underlying MM or B-CLL. In addition, we observed low frequency of unbalanced serum FLCr in the SID and UAD groups, respectively 3 and 12% of subjects.

When analyzing immunoglobulin serum levels at diagnosis in the different cohorts, IgA levels were directly associated with serum κ and λ chain concentrations in CVID (*R*^2^ 0.03 *p* < 0.0001 and *R*^2^ 0.07 *p* < 0.0001, respectively) and UAD (*R*^2^ 0.25, *p* < 0.0001, *R*^2^ 0.25, *p* < 0.0007), but not in LPDs and SID (Figures S1A,B). Only in the CVID cohort IgM was directly associated with both serum κ and λ chain concentrations (*R*^2^ 0.46 *p* < 0.0001 and *R*^2^ 0.05 *p* < 0.0001, respectively—Figures S2A,B). No association was found between sFLC serum concentration and IgG in CVID and in control groups.

### sFLC Pattern

**Figure 2:**
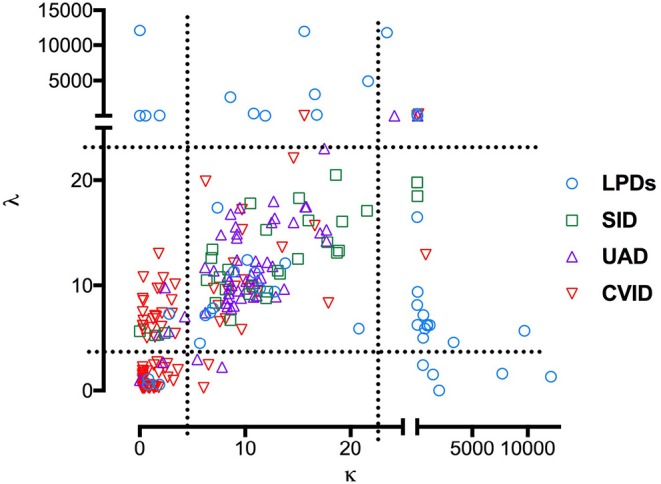
Combined sFLC levels in the differential diagnosis of hypogammaglobulinemia. The scatter plot shows sFLC levels for 345 patients with CVID (red triangles), UAD (purple triangles), LPDs (blue circles) and non-hematological SID (green squares). Dashed lines represent reference for sFLC values.

Based on sFLC reference values, patients were grouped into four phenotypes (Figure 2). For CVID, the most common pattern was k–λ– (51%), followed by k–λ+ (25%), k+λ+ (22%), and k+λ– (3%). Differently from CVID, in the UAD, LPD, and SID groups, the κ+λ+ was the most common pattern observed, including, respectively 83 (*p* < 0.0001), 70 (*p* < 0.0001), and 90% (*p* < 0.0001) of patients. LPD patients showed more commonly very high (>150 mg/L) concentration of at least one sFLC in comparison to CVID (45 vs. 0.6%, *p* < 0.0001). In the CVID, in addition to the above-mentioned patients who developed NHL, we recorded high concentration of both κ and λ chains in one patient with balanced FLCr and recurrent autoimmune cytopenia and chronic lymphadenopathy. None in the UAD and in the SID groups had very high concentration of κ or λ chains (Figure 2).

### Sensitivity, Specificity, and Positive Predictive Value (PPV) of sFLC Pattern in CVID

Since 79% of CVID patients had at least one reduced FLC isotype but none of them had increased concentration of the other κ or λ chain, we defined as *CVID-like sFLC phenotype* the condition of having at least one reduced sFLC isotype without an increase of either κ or λ chain concentration, as previously suggested (15). In this cohort, sensitivity of the CVID-like pattern of sFLC was 78.3% (95% CI, 73.5 to 82.5%), and specificity was 87.6% (95% CI, 80.9 to 92.6%). By using this pattern as a diagnostic marker for CVID, the positive predictive value was 90.1% (95% CI, 91.0 to 96.1%), and the negative predictive value was 61.5% (95% CI, 56.5 to 66.4%).

### Association Between sFLC Patterns and Clinical Phenotype in CVID Patients

Age at the time of enrollment did not significantly differ in CVID groups when considering the sFLC pattern. However, κ+λ+ patients were older at CVID diagnosis (45 y.o. IQR 32–45) in comparison to κ−λ− (38 y.o., IQR 32–45, *u*-test *p* = 0.001) and κ−λ+ group (39 y.o., IQR 26–42, *u*-test *p* = 0.020).

**Figure 3:**
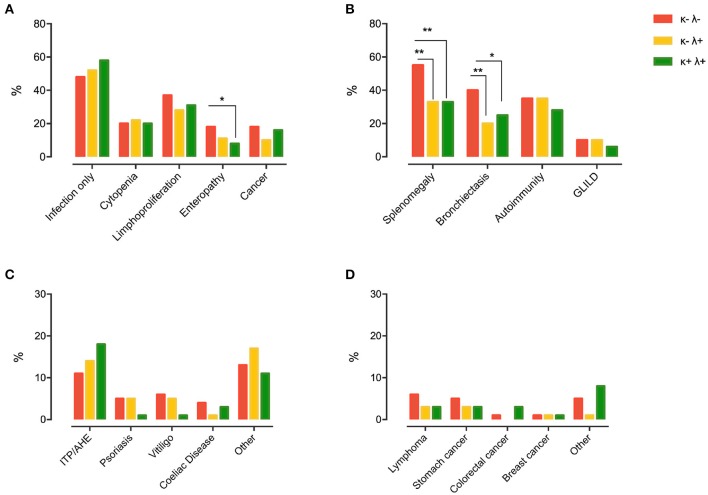
Chapel clinical phenotyping **(A)** and CVID-related comorbidities **(B)** in participants grouped by FLC pattern (κ+λ− group was censored as it was composed of only nine subjects). The most prevalent types of autoimmune diseases and cancers in the different CVID subgroups are detailed in **(C,D)**, respectively. Levels of significance for comparison by Fisher's test: *P ≤ 0.05, **P ≤ 0.01. ITP, idiopathic thrombocytopenic purpura; AHE, autoimmune hemolytic anemia; GLILD, granulomatous and lymphocytic interstitial lung disease.

When analyzing the possible correlations between sFLC patterns and disease-related clinical phenotype and complications in CVID patients, the only disease phenotype significantly associated with the sFLC pattern was the “enteropathy” phenotype, with patients belonging to the κ−λ− group showing a higher frequency of enteropathy than those classified as κ+λ+ (18 vs. 8%, *p* = 0.050) (Figure 3A). In comparison to those included in the κ+λ+ and κ−λ+ group, κ−λ− patients were also more likely to have bronchiectasis (40 vs. 25%, *p* = 0.030 and vs. 20%, *p* = 0.002, respectively) and splenomegaly (55 vs. 33%, *p* = 0.002 and vs. 33%, *p* = 0.001, respectively) (Figure 3B). A medical history consistent with autoimmunity, cancer, lymphoma, or GLILD was not found to be associated with FLC patterns (Figures 3A–D).

### Association Between sFLC Patterns and Laboratory Signatures of CVID

**Figure 4:**
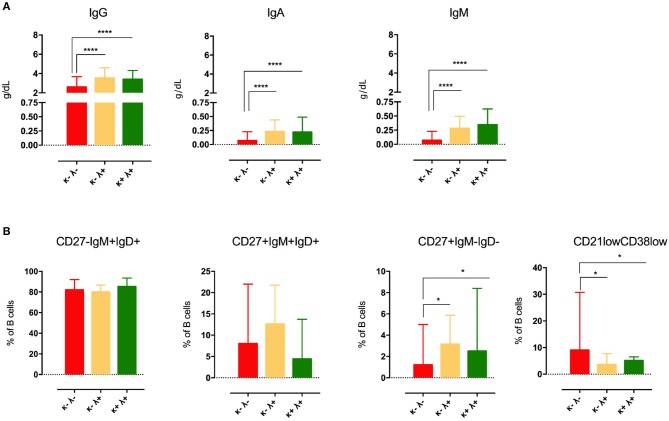
Immunoglobulin serum levels at CVID diagnosis **(A)** and B-cell phenotype **(B)** in CVID patients grouped by sFLC levels (κ+λ− group was censored as it was composed of only nine subjects). Median and IQR are represented. Levels of significance for comparison with CVID group by u test: *P ≤ *0.05*, ****P ≤ 0.0001.

As shown in Figure 4A, the κ−λ− group showed lower IgG (2.6 g/L, IQR 1.4–3.7), IgA (0.1 g/L, IQR 0.1–0.2), and IgM serum levels (0.1 g/L, IQR 0.0–0.2 g/L) in comparison to κ−λ+ (IgG 3.5 g/L, IQR 2.3–4.6, *u*-test *p* < 0.0001; IgA 0.2 g/L, IQR 0.1–0.4, *u*-test *p* < 0.0001; IgM 0.3 g/L, IQR 0.1–0.5, *u*-test *p* < 0.0001), and κ+λ+ patients (IgG 3.4 g/L, IQR 2.8–4.3, *u*-test *p* = 0.012; IgA 0.2 g/L, IQR 0.1–0.5, *u*-test *p* < 0.0001, IgM 0.3 g/L, IQR 0.2–0.6, *u*-test *p* < 0.0001). κ−λ− CVID patients also presented lower frequency of CD27+IgD–IgM– Switched Memory B cells (1.2%, IQR 0.4–5.0) when compared to both κ−λ+ and κ+λ+ groups (3.5%, IQR 1.0–5.9, *u*-test *p* = 0.050 and 2.5%, IQR 1.7–8.4, *u*-test *p* = 0.027, respectively) and the highest frequency of CD21^low^ B cells among the CVID subgroups, with a significant difference in comparison to both κ−λ+ and κ+λ+ patients (9.0%, IQR 3.1–31.0 vs. 3.6, IQR 2.2–7.7, *u*-test *p* = 0.013, and *vs* 5.1%, IQR 1.9–6.5, *u*-test *p* = 0.030) (Figure 4B).

## Discussion

To our knowledge, this is the first multicenter study exploring the diagnostic value and the clinical association of sFLC pattern in a large cohort of CVID patients. Patients were recruited all over Italy, in six PAD referral centers. sFLC assay was performed in a single laboratory according to standard diagnostic procedure. As control groups, we enrolled subjects selected on the basis of a referral for hypogammaglobulinemia and finally diagnosed as UAD patients and secondary hypogammaglobulinemia, respectively. This latter group was further divided, on the basis of the final diagnosis, into a lymphoproliferative disease group (LPD), including MM, B-CLL, Non-Hodgkin Lymphomas and MGUS, and a secondary immunodeficiency group (SID) whose hypogammaglobulinemia was not related to LPDs.

In agreement with previous observations, we found that CVID patients tend to have a very low sFLC count with 79% of them presenting a significant reduction in one (almost exclusively κ) or both (κ and λ) chains, often to an undetectable level. All CVID patients were on IgRT; however, as previously shown, regular immunoglobulin administration did not affect the circulating sFLC concentrations (14–16). A very good intraindividual agreement between different sFLC assays was also registered.

Less than a half (41%) of the CVID patients presented an abnormal FLCr; for almost all of them this was the result of a decreased concentration of one chain with no increase in the other, therefore not consistent with a B cell malignancy. On the contrary, most patients (82%) in the LPD group showed an unbalanced FLCr due to an increase in either κ or λ chain, which was related to an underlying MM or B-CLL. In the SID and UAD groups a minimal percentage of unbalanced FLCr (3 and 12%, respectively) and no increase in κ or λ chain concentration were observed.

As suggested by Unsworth et al. our study supports the idea that in CVID patients, an impaired κ/λ ratio due to very low concentration of at least one sFLC most likely underlies the disease-related B-cell dysfunction, rather than a B lymphocyte clonality (14). On the other hand, the detection of an unbalanced sFLCr with a single light chain increase in two CVID patients from our cohort led to a diagnosis of a lymphoproliferative disease. One of these two patients, in particular, initially presented a reduction of both light chains and subsequently developed a significant increase of circulating lambda chain, with no detectable paraprotein and was eventually diagnosed a NHL. This suggests that sFLC assay might also be an effective tool in the follow-up strategy in a population with a well-known risk of hematological malignancies (4, 24, 25).

The overall percentage of CVID patients with at least one reduced light chain was similar to that reported by Compagno et al. (88%) and higher than described by Hanitsch et al. (63%). The so-called CVID-like sFLC pattern presented a sensitivity of 78.3% and a specificity of 87.6% and, as a diagnostic marker for CVID, a positive predictive value of 90.1% and a negative predictive value of 61.5% (15, 16). In the control groups the percentage of patients with at least one reduced light chain was 17, 10, and 30% for UAD, SID, and LPDs, respectively, with most of LPD patients showing a monoclonal increase in the other chain. In agreement with previous reports, our results confirm sFLC assay as a highly sensitive and specific tool in the diagnostic work-up of CVID that might be combined with other existing B cell assays. By considering also the SID group, not included in previous studies, we confirmed its reliability in “real life” differential diagnosis.

In our multicenter CVID cohort, the distribution of the four different patterns of sFLCs, normal kappa, and lambda chains (κ+λ+), reduced kappa chains (κ−λ+), reduced lambda chains (κ+λ−), and reduction of both chains (κ−λ−) confirms what was reported in our previous single-center study, and it shows similarities with another recently published study (15, 16). The more represented pattern in our cohort was indeed the (κ−λ−), with only few patients belonging to the (κ+λ−) group. In contrast to the paper by Hanitsch et al. we found that the number of patients in the (κ−λ+) group was more relevant, being slightly higher than the (κ+λ+) group.

The association of sFLC pattern with clinical data showed that CVID patients belonging to the κ−λ− group were more frequently classified as having unexplained enteropathy. We did not observe significant differences in terms of autoimmunity, GLILD, and cancer between the three groups. Moreover, κ−λ− patients presented more commonly bronchiectasis as described by Hanitsch et al. and splenomegaly as reported by Compagno et al. in comparison to those included in the κ−λ+ and κ+λ+ groups. Considering the infectious risk, when compared to the other groups, κ−λ− patients also showed lower serum IgG, IgA, and IgM levels at diagnosis, a profile that may predict a higher risk for bacterial infections and chronic lung damage (26). Thus, our findings point out to a more severe infectious phenotype as the reason for the earlier diagnosis of CVID reported in κ−λ− patients. Moreover, we found a direct association between serum IgA levels and serum κ and λ chain concentrations in CVID and UAD cohorts (but not in LPDs and SID) and, in CVID only, a direct association between serum IgM and both serum κ and λ chain concentrations, further strengthening the correlation between sFLC levels and immunoglobulin production suggested by Unsworth et al. (14).

When considering circulating B cell subpopulations according to the EUROclass trial, we did not observe any difference in CD19+, transitional and marginal-zone B cells among the three main subgroups of CVID patients (20). However, κ−λ− CVID patients presented lower frequency of CD27+IgD–IgM– Switched Memory B cells when compared both to the κ−λ+ and κ+λ+ groups. This is consistent with the hypothesis that lower levels of sFLC may be an epiphenomenon of a higher degree of impairment in B cell differentiation, with a reduced B cell isotype switch leading to a lower level of immunoglobulin production. κ−λ− patients also showed the highest frequency of CD21^low^ B cells between the CVID subgroups, with a significant difference if compared to both κ−λ+ and κ+λ+ patients. Of note, reduction in switched memory and increase in CD21^low^ B cells, history of autoimmune cytopenia, presence of splenomegaly, and low serum IgA levels have been all suggested as potential predictors of GLILD (27, 28). According to the sFLC pattern, in our study κ−λ− patients present all the above-mentioned predictors apart from the history of autoimmune cytopenia. In contrast with Hanitsch et al. however, κ−λ− patients did not show significantly higher frequency of GLILD diagnosis, when compared to the other CVID subgroups (16). This difference might be partly due to the lack of universally recognized diagnostic criteria for GLILD, or simply to the different behaviors in case of GLILD suspicion at chest CT scan, particularly in asymptomatic patients. The choice between a more invasive approach with histologic confirmation and a watchful waiting strategy may indeed lead to a different degree of definite diagnoses. The ongoing prospective study in our cohort will hopefully grant more information on sFLC pattern as a possible long-term predictor of GLILD.

In conclusion, sFLC assay may be reasonably considered as a useful tool to be included in the differential diagnosis of antibody deficiencies, with a good specificity and sensitivity for CVID. Due to its well-known role in diagnosis and prognosis of LPDs, and based on our and previously published data, we suggest adding sFLC dosage to the initial screening of hypogammaglobulinemia and during follow-up. The assay may help in distinguishing CVID from other causes of hypogammaglobulinemia, in combination with other tests, and it offers the chance of early detection of monoclonal lymphoproliferation occurring over years. Moreover, the sFLC pattern appears to be related to other laboratory parameters and to disease phenotypes and clinical manifestations of CVID. If our data will be confirmed by further and long-term follow-up studies, sFLC assay will thus be considered a useful prognostic tool that, in combination with other instruments, might help in identifying disease phenotypes at higher risk to develop enteropathy and chronic lung damage or splenomegaly and further complications, thus designing a more personalized follow-up strategy for CVID patients.

## Data Availability Statement

The datasets generated for this study are available on request to the corresponding author.

## Ethics Statement

The studies involving human participants were reviewed and approved by Comitato Etico per la Sperimentazione Clinica della Provincia di Padova. The patients/participants provided their written informed consent to participate in this study.

## Author Contributions

RS and FP wrote the paper and contributed to patient enrolment and data collection. FP did the statistical analysis. FC and CMi equally contributed to designing and coordinating the study, were involved in patient enrolment and data collection, manuscript writing, and revision (joint authorship). CA, IQ, GS, AM, AVa, and MD contributed to designing the study, were involved in data analysis, and revised the paper. NC contributed to designing the study and to patient enrolment. AP, CMa, AVu, FV, and RR contributed to patient enrollment, data collection and paper revision. DF contributed to data interpretation, and manuscript preparation and revision. SA performed the sFLC assay and contributed to manuscript preparation and revision. MP contributed to manuscript preparation and revision.

### Conflict of Interest

The authors declare that the research was conducted in the absence of any commercial or financial relationships that could be construed as a potential conflict of interest.

## Supplementary Material

Figure S1 Linear correlation between IgA serum levels at diagnosis and serum κ **(A)** and λ **(B)**. IgA levels were directly associated with free κ and λ values in CVID and UAD, but not in LPDs and SID.

Figure S2 Linear correlation between IgM serum levels at diagnosis and serum κ **(A)** and λ **(B)**. Only in the CVID cohort IgM was directly associated with both serum κ and λ chain concentrations. No association was found between sFLC and IgM levels at diagnosis in the control groups.
