# Supplementary material for: Serum Free Light Chains in Common Variable Immunodeficiency Disorders: Role in Differential Diagnosis and Association With Clinical Phenotype
Source: Front Immunol. 2020 Mar 31;11:319. doi: 10.3389/fimmu.2020.00319 (PMC7136404; doi:10.3389/fimmu.2020.00319)
Supplement: Supplementary file 1 [file Table_1.docx]

**SUPPLEMENTARY TABLE 1.** Final diagnosis for patients classified as having hypogammaglobulinemia due to LPDs or SID.

| ID | Diagnosis | κ  (mg/L) | λ  (mg/L) | IgG  (mg/L) | IgA  (mg/L) | IgM  (mg/L) |
| --- | --- | --- | --- | --- | --- | --- |
| **LPDs** |  |  |  |  |  |  |
| 346 | B-CLL | <0,35 | 27,9 | 1,68 | 0,35 | 0,76 |
| 347 | B-CLL | 10,2 | 12,4 | 5,19 | 0,53 | 0,23 |
| 348 | B-CLL | 11,9 | 54,3 | 6,93 | 0,09 | 0,17 |
| 349 | B-CLL | 28,6 | 8,14 | 5,76 | 0,31 | 0,35 |
| 350 | B-CLL | 8,94 | 11,3 | 6,88 | 0,16 | 1,28 |
| 351 | B-CLL | 72,9 | 9,38 | 3,96 | 0,12 | 0,05 |
| 352 | CM | 2,8 | 7,3 | 5,5 | 0,46 | 1,26 |
| 353 | LDGL | 13,8 | 12,1 | 9,04 | 1,22 | 0,56 |
| 354 | LDGL | 13,1 | 11,4 | 5,7 | 1,4 | 1,1 |
| 355 | B-CLL | 0,77 | 0,56 | 4,29 | 0,28 | 0,05 |
| 356 | B-CLL | 1,88 | 77,2 | 5,46 | 0,48 | 0,05 |
| 357 | B-CLL | 0,82 | 1,09 | 4,63 | 1,94 | 0,17 |
| 358 | NHL (follicular B cells lymphoma) | 11,4 | 9,72 | 4,48 | 1,44 | 0,73 |
| 359 | NHL (marginal zone B cells lymphoma) | 6,93 | 7,83 | 4,17 | 2,43 | 0,17 |
| 360 | MBL with B-CLL phenotype | 10,9 | 11,2 | 8,6 | 1,51 | 0,36 |
| 361 | MGUS | 59,7 | 38,3 | 14 | 0,16 | 0,12 |
| 362 | MGUS | 12,8 | 9,4 | 5,43 | 1,73 | 0,4 |
| 363 | MGUS | 6,69 | 7,42 | 6,17 | 0,56 | 0,38 |
| 364 | MGUS | 5,7 | 4,5 | 6,51 | 1,32 | 1,03 |
| 365 | MGUS | 7,38 | 17,4 | 5,31 | 3,17 | 0,31 |
| 366 | MGUS | 37,4 | 16,5 | 6,05 | 2,82 | 0,38 |
| 367 | MGUS | 19,2 | 16,1 | 2,95 | 0,8 | 0,44 |
| 368 | MGUS | 6,23 | 7,17 | 5,39 | 0,4 | 0,34 |
| 369 | MGUS | 34 | 293 | 4,59 | 1,77 | 0,56 |
| 370 | MGUS | 20,8 | 5,9 | 5,72 | 1,24 | 0,68 |
| 371 | MM | 948 | 6,2 | 5,55 | 0,19 | 0,05 |
| 372 | MM | 9710 | 5,7 | 1,75 | 0,08 | 0,05 |
| 373 | MM | <0,35 | 12100 | 2 | 0,19 | 0,05 |
| 374 | MM | 8,61 | 2650 | 4,62 | 1,03 | 0,26 |
| 375 | MM | 0,55 | 35 | 2,15 | 0,23 | 0,55 |
| 376 | MM | 18900 | 10,9 | 2,56 | 0,06 | 0,16 |
| 377 | MM | 23,5 | 11800 | 6,9 | 0,28 | 0,17 |
| 378 | MM | 579 | 7,19 | 4,58 | 0,23 | 0,22 |
| 379 | MM | 15,6 | 12000 | 5,83 | 0,25 | 0,09 |
| 380 | MM | 21,7 | 4920 | 5,2 | 0,38 | 0,05 |
| 381 | MM | 16,6 | 3060 | 5,42 | 0,88 | 0,19 |
| 382 | MM | 948 | 6,29 | 6,19 | 0,19 | 0,05 |
| 383 | MM | 10,8 | 338 | 5,31 | 0,44 | 0,8 |
| 384 | MM | 12100 | 1,32 | 4,2 | 0,18 | 0,08 |
| 385 | MM | 1450 | 1,53 | 6,55 | 0,37 | 0,17 |
| 386 | MM | 2020 | <0,35 | 3,08 | 0,22 | 0,26 |
| 387 | MM | 7700 | 1,6 | 5,81 | 0,2 | 0,05 |
| 388 | MM | 540 | 2,41 | 4,2 | 0,53 | 0,43 |
| 389 | MM (micromolecular) | 16,8 | 168 | 4,21 | 1,35 | 1,26 |
| 390 | MM (micromolecular) | 732 | 5,9 | 4,42 | 0,26 | 0,22 |
| 391 | MM (micromolecular) | 1170 | 6,24 | 3,29 | 0,13 | 0,16 |
| 392 | MM (micromolecular) | 3310 | 4,59 | 5,8 | 0,67 | 0,05 |
| 393 | MM (smoldering) | 45,4 | 6,27 | 3,78 | 11,6 | 0,3 |
| 394 | MM (smoldering) | 568 | 5 | 3,4 | 0,9 | 0,56 |
|  |  |  |  |  |  |  |
| **SID** |  |  |  |  |  |  |
| 395 | post CCS hypogammaglobulinemia (CKD, AIHE) | 40,4 | 19,8 | 4,55 | 2,48 | 0,39 |
| 395 | post CCS hypogammaglobulinemia (COPD) | 10,5 | 17,8 | 7,32 | 1,68 | 1,34 |
| 396 | post CCS hypogammaglobulinemia (CM) | 47,1 | 18,5 | 5,9 | 1,3 | 0,55 |
| 397 | miotonic dystrofia | 7,19 | 8,35 | 5,95 | 1,21 | 1,43 |
| 398 | post CCS hypogammaglobulinemia (nephrosic syndrome) | 12 | 15,3 | 3,66 | 1,12 | 0,16 |
| 399 | post CCS and DMARs hypogammaglobulinemia | 8,15 | 9,61 | 6,72 | 2,31 | 0,75 |
| 400 | transitory post infective hypogammaglobulinemia | 6,8 | 12,6 | 5,97 | 1,84 | 0,89 |
| 401 | post CCS hypogammaglobulinemia (asthma) | 10,4 | 9,18 | 6,58 | 1,71 | 0,32 |
| 402 | post CCS hypogammaglobulinemia | 8,59 | 6,72 | 5,05 | 0,47 | 0,36 |
| 403 | transitory hypogammaglobulinemia | 7,59 | 10,8 | 5,83 | 1,48 | 0,42 |
| 404 | protein losing enteropathy | 34,9 | 19,8 | 6,75 | 1,22 | 0,23 |
| 405 | post CCS hypogammaglobulinemia | 13,3 | 11,1 | 6,73 | 1,71 | 0,38 |
| 406 | post CCS hypogammaglobulinemia (RA) | 18,6 | 20,5 | 5,77 | 1,22 | 1,37 |
| 407 | transitory post infective hypogammaglobulinemia | 8,4 | 11,5 | 7,79 | 2,43 | 0,22 |
| 408 | post CCS hypogammaglobulinemia (rheumatic polymyalgia, CKD) | 16 | 16,2 | 4,11 | 0,46 | 0,28 |
| 409 | transitory post infective hypogammaglobulinemia | 12 | 8,79 | 7,46 | 2,1 | 1,01 |
| 410 | transitory infective hypogammaglobulinemia | 17,8 | 14,1 | 7,41 | 1,59 | 1,11 |
| 411 | post parkinson treatment hypogammaglobulinemia | 12,1 | 9,4 | 5,74 | 0,8 | 0,68 |
| 412 | SS, PBC, Sjogren syndrome | 15,1 | 18,3 | 5,73 | 1,17 | 1,09 |
| 413 | post CCS hypogammaglobulinemia | 18,9 | 13,3 | 6,15 | 1,4 | 0,68 |
| 414 | CKD | 18,7 | 13,1 | 3,98 | 0,94 | 0,13 |
| 415 | post CCS hypogammaglobulinemia (MS) | 10,7 | 9,9 | 4,62 | 1,17 | 1,43 |
| 416 | post CCS hypogammaglobulinemia (MS) | 6,9 | 13,4 | 6,04 | 2,08 | 2,05 |
| 417 | post RTX hypogammaglobulinemia (AIHE, ITP) | 21,6 | 17,1 | 5,32 | 1,87 | 0,17 |
| 418 | transitory hypogammaglobulinemia | 6,35 | 10,5 | 6,44 | 1,28 | 0,55 |
| 420 | myelodysplastic syndrome | 15 | 12,5 | 6,82 | 1,17 | 0,32 |
| 422 | post RTX hypogammaglobulinemia (nephrosic syndrome and CM) | 2,37 | 5,5 | 5,82 | 0,61 | 0,34 |
| 423 | post RTX hypogammaglobulinemia (SLE) | <0,35 | 5,66 | 3,38 | 0,08 | 0,05 |
| 424 | post CCS hypogammaglobulinemia | 1,46 | 5,24 | 6,36 | 0,78 | 0,5 |

**SID**: 14 males, 15 females; mean age 67.2 ± 11.2 years. **LPDs**: 30 Males, 18 Females; mean age 56.1 ± 14.9 years. Abbreviations: AIHE, autoimmune hemolytic anemia; CCS, corticosteroids; CLL, chronic lymphocytic leukemia; CM, mixed cryoglobulinemia; CKD, chronic kidney disease; COPD, chronic obstructive pulmonary disease; ITP, idiopathic thrombocytopenic purpura; LDLG, T-cell type lymphoproliferative disease of granular lymphocytes; MBL, monoclonal B cell lymphocytosis; MM, multiple myeloma; MGUS, monoclonal gammopathy of unknown significance; MS, multiple sclerosis; NHL, non-Hodgkin lymphoma; RA, rheumatoid arthritis; RTX, rituximab; SS, systemic sclerosis
