# Supplementary figures and images for: Serum Free Light Chains in Common Variable Immunodeficiency Disorders: Role in Differential Diagnosis and Association With Clinical Phenotype
Source: Front Immunol. 2020 Mar 31;11:319. doi: 10.3389/fimmu.2020.00319 (PMC7136404; doi:10.3389/fimmu.2020.00319)

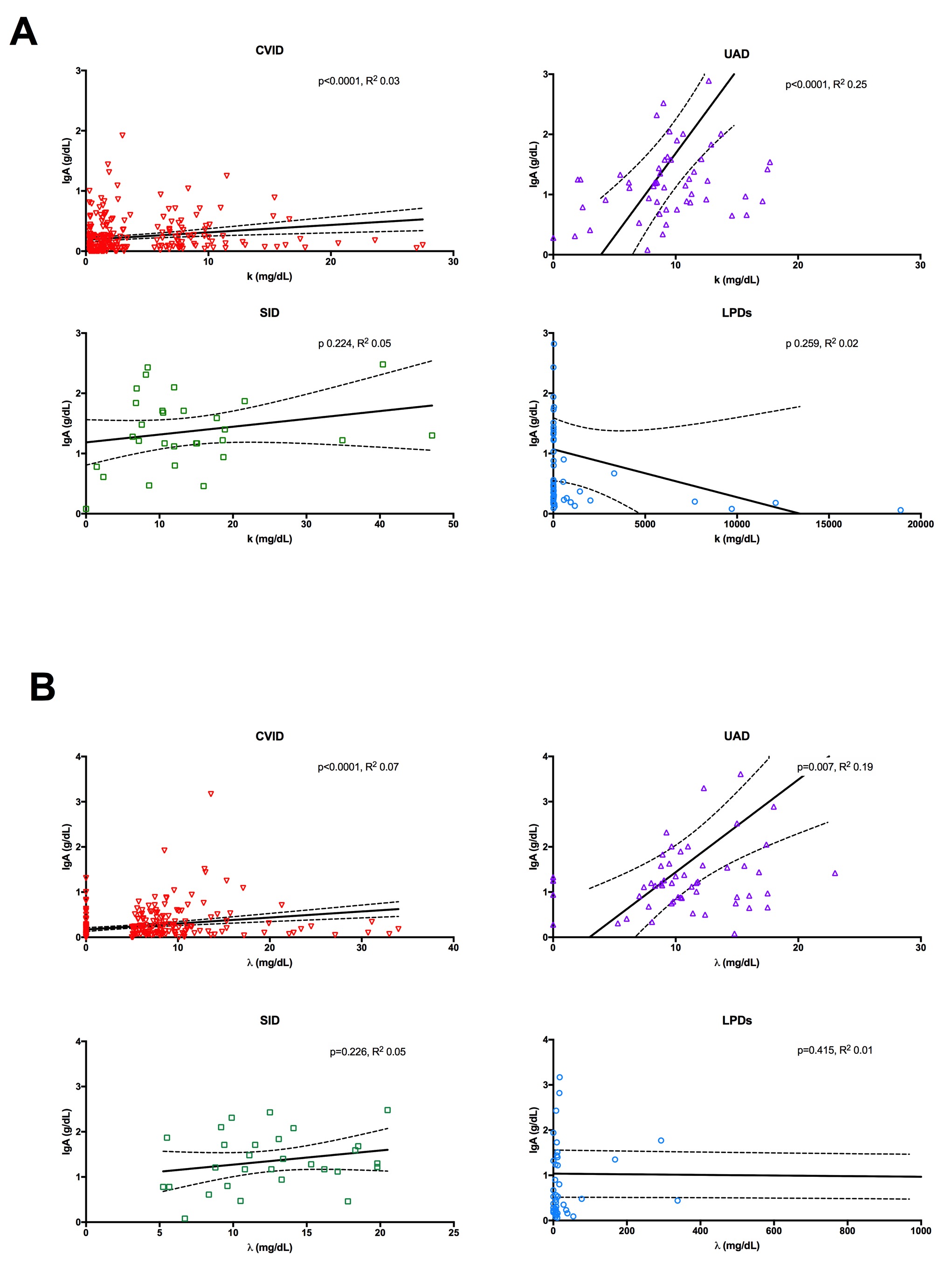

Supplement: Figure S1 — Linear correlation between IgA serum levels at diagnosis and serum κ (A) and λ (B). IgA levels were directly associated with free κ and λ values in CVID and UAD, but not in LPDs and SID. [file Image_1.jpg]

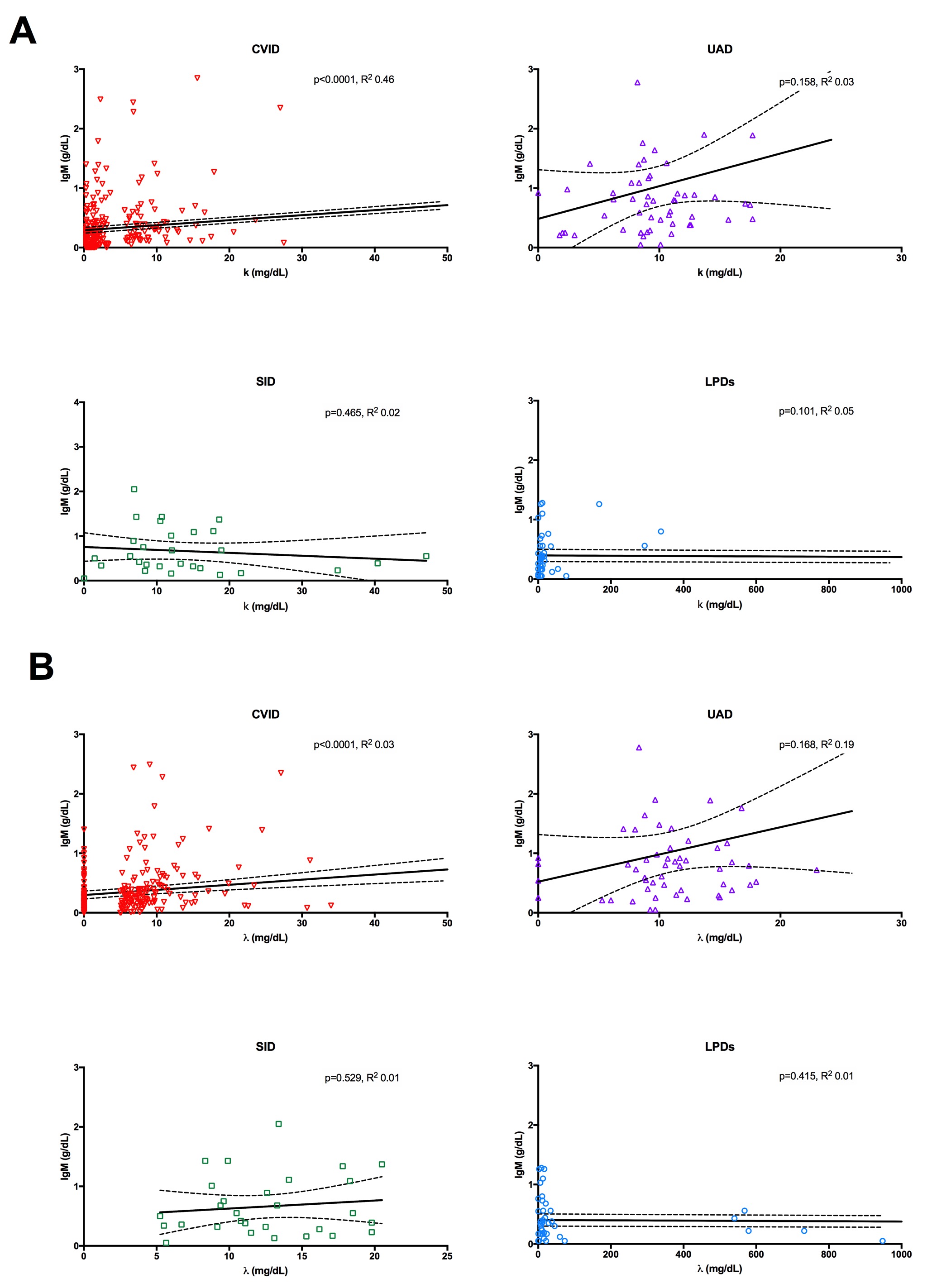

Supplement: Figure S2 — Linear correlation between IgM serum levels at diagnosis and serum κ (A) and λ (B). Only in the CVID cohort IgM was directly associated with both serum κ and λ chain concentrations. No association was found between sFLC and IgM levels at diagnosis in the control groups. [file Image_2.jpg]
